# Natural Competence in the Filamentous, Heterocystous Cyanobacterium *Chlorogloeopsis fritschii* PCC 6912

**DOI:** 10.1128/msphere.00997-21

**Published:** 2022-07-14

**Authors:** Fabian Nies, Benjamin L. Springstein, Dustin M. Hanke, Tal Dagan

**Affiliations:** a Institute of General Microbiology, Christian-Albrechts-Universität zu Kiel, Kiel, Germany; University of Illinois at Urbana-Champaign

**Keywords:** natural transformation, lateral gene transfer, genome evolution, cyanobacteria

## Abstract

Lateral gene transfer plays an important role in the evolution of genetic diversity in prokaryotes. DNA transfer via natural transformation depends on the ability of recipient cells to actively transport DNA from the environment into the cytoplasm, termed natural competence, which relies on the presence of type IV pili and other competence proteins. Natural competence has been described in cyanobacteria for several organisms, including unicellular and filamentous species. However, natural competence in cyanobacteria that differentiate specialized cells for N_2_-fixation (heterocysts) and form branching or multiseriate cell filaments (termed subsection V) remains unknown. Here, we show that genes essential for natural competence are conserved in subsection V cyanobacteria. Furthermore, using the replicating plasmid pRL25C, we experimentally demonstrate natural competence in a subsection V organism: *Chlorogloeopsis fritschii* PCC 6912. Our results suggest that natural competence is a common trait in cyanobacteria forming complex cell filament morphologies.

**IMPORTANCE** Cyanobacteria are crucial players in the global biogeochemical cycles, where they contribute to CO_2_- and N_2_-fixation. Their main ecological significance is the primary biomass production owing to oxygenic photosynthesis. Cyanobacteria are a diverse phylum, in which the most complex species differentiate specialized cell types and form true-branching or multiseriate cell filament structures (termed subsection V cyanobacteria). These bacteria are considered a peak in the evolution of prokaryotic multicellularity. Among others, species in that group inhabit fresh and marine water habitats, soil, and extreme habitats such as thermal springs. Here, we show that the core genes required for natural competence are frequent in subsection V cyanobacteria and demonstrate for the first time natural transformation in a member of subsection V. The prevalence of natural competence has implications for the role of DNA acquisition in the genome evolution of cyanobacteria. Furthermore, the presence of mechanisms for natural transformation opens up new possibilities for the genetic modification of subsection V cyanobacteria.

## OBSERVATION

DNA acquisition by lateral transfer plays a major role in the evolution of prokaryotic organisms. Recombination at the species level contributes to selective sweeps through the population, while interspecies lateral gene transfer has important implications to microbial adaptation and evolutionary innovations. The commonly known mechanisms of DNA transfer in bacteria include conjugation, transduction, and transformation (reviewed in [1]). DNA transfer via conjugation and transduction is mediated by mobile genetic elements, which are mobile or mobilizable plasmids, or phages. In contrast, DNA acquisition via transformation depends only on the ability of the recipient cell to actively transport DNA from the environment into the cytoplasm, which is termed natural competence (2). Natural competence was shown in several unicellular, subsection I cyanobacteria, including the model organisms *Synechocystis* spp. (3, 4) and *Synechococcus* spp. (5, 6), as well as the thermophilic Thermosynechococcus elongatus BP-1 (7) and the toxic bloom-forming Microcystis aeruginosa PCC 7806 (8). Natural competence has also been described in three filamentous cyanobacteria: the heterocystous (able to differentiate some cells within a cell filament into heterocysts, specialized cells types for N_2_-fixation), subsection IV cyanobacterium Nostoc muscorum (9), and, more recently, Phormidium lacuna HE10DO (10), a subsection III, nonheterocystous, marine cyanobacterium, and the commercially relevant *Arthrospira platensis* (11).

Natural competence in most gram-negative bacteria relies on type IV pili, which are also involved in motility, adhesion, and protein secretion (12). Additionally, the competence proteins ComEA, ComEC, and ComF are essential for DNA uptake into the cell (2, 13). Proteins binding single-stranded DNA, including the DNA processing protein DprA and the DNA recombination and repair protein RecA, are required for subsequent integration of the acquired DNA into the recipient genome (13). The experimental investigation of proteins essential for natural competence in cyanobacteria has been focused mainly on the unicellular model organisms *Synechocystis* sp. PCC 6803 and Synechococcus elongatus PCC 7942. Studies of both species revealed several core genes that are essential for natural competence, including *comEA*, *comEC*, *comF*, *dprA*, *pilA1*, *pilB1*, *pilC*, *pilD*, *pilM*, *pilN*, *pilO*, *pilQ*, *pilP*, and *pilT1* (14–18), which are also known to be essential for natural competence in other gram-negative bacteria (2, 13). Recent studies further revealed that the RNA chaperone Hfq and biofilm suppressing EbsA interact with PilB1 and are essential for pilus formation and, thus, for natural transformation in those two cyanobacteria (19, 20). Additionally, previous studies have assessed the distribution of genes essential for natural competence in several cyanobacterial genomes and those studies suggest that natural competence is a prevalent trait within the phylum (10, 17, 21). Nonetheless, studies on natural competence in subsection V cyanobacteria have not been conducted.

Here, we investigate the presence of natural competence genes in subsection V cyanobacteria. Species classified in this group grow as multicellular cell filaments that differentiate heterocysts, spore-like resting cells (akinetes), and motile cell filaments (hormogonia) (22). The hallmark trait of subsection V cyanobacteria is their complex multicellular morphology that includes true-branching cell filaments (as in *Fischerella* species) and multiseriate cell filaments (as in *Chlorogloeopsis* species). The morphological diversity and the multiplicity of differentiated cell types of subsection V cyanobacteria place them among the most complex prokaryotes.

**FIG 1:**
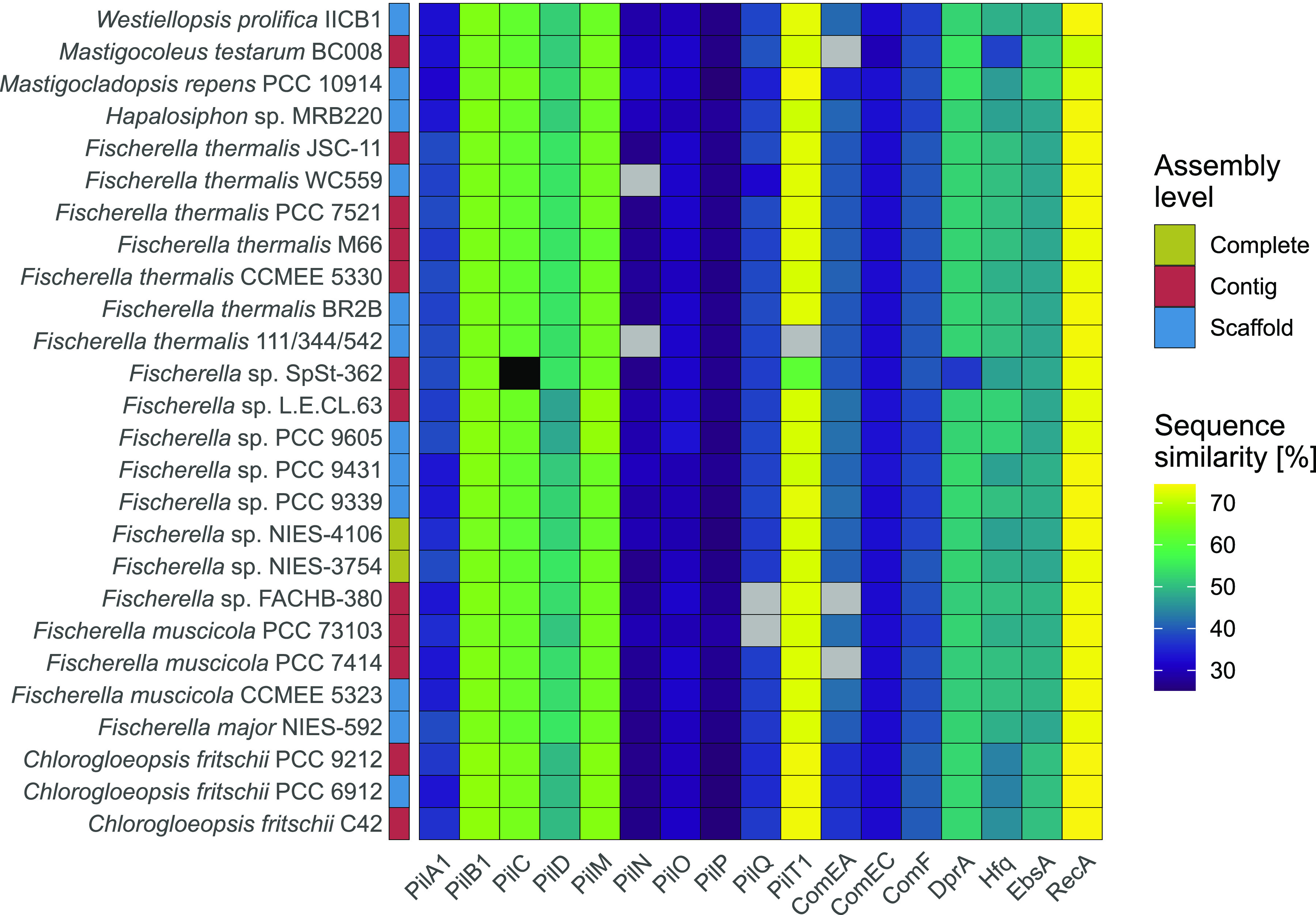
Distribution of homologs of the core natural competence genes in subsection V cyanobacteria. Cells in the matrix are colored according to the percent sequence similarity (see color-bar on the right) between the *Synechocystis* sp. PCC 6803 query protein sequence and the respective subsection V cyanobacterium homologs (strain name shown on the left). The assembly level of the subsection V cyanobacteria genome is given in the left column. Gray cells correspond to missing hits on the protein level, but by tBLASTn, similar sequences can be found (>70% query coverage) on the genomes. Missing annotation of these proteins can correspond to deleterious single nucleotide polymorphisms (SNPs). These SNPs can either have biological relevance or be caused by suboptimal sequencing quality. For the black cell, no specific hits were found by tBLASTn (see Data Set S1 for further information on sequence similarity and additional strains). The level of protein sequence conservation toward the *Synechocystis* sp. PCC 6803 query proteins differs among the investigated genes. PilT, an ATPase responsible for pilus retraction, is nearly as conserved as RecA, a protein considered to be nearly ubiquitous in bacteria (38). Also, PilB, the antagonistic ATPase for pilus elongation, as well as the pilus components PilC and PilM, the prepilin peptidase PilD, and DprA, Hfq, and EbsA all share a sequence similarity of around 50% or higher with the query protein sequence. The sequences of other parts of the pilus and the competence proteins ComEA, ComEC, and ComF are less conserved.

To predict naturally competent cyanobacteria of subsection V, we surveyed for homologs to the above-mentioned core natural competence genes (NCGs) from *Synechocystis* sp. PCC 6803 (Table S1) in subsection V cyanobacteria genomes. The distribution of NCG homologs in the genomes of subsection V cyanobacteria shows that most tested species harbor the core NCGs (Fig. 1) and, hence, could potentially be naturally competent. Within subsection V cyanobacteria, the proteins encoded by NCGs are highly conserved, with a mean pairwise identity ranging from 60% for PilA1 up to 95% for RecA (Fig. S1). Several strains of *Fischerella* and Mastigocoleus testarum BC008 are lacking at least one NCG protein-coding sequence. In most of these cases, the missing NCGs could be manually identified by searching the DNA genome sequence (Fig. 1); however, the missing NCG annotation might indicate that these genes are nonfunctional (i.e., pseudogenes). The presence of the complete set of NCGs in other *Fischerella* species in our analysis suggests that the potential nonfunctionalization of these pseudogenes might have occurred rather recently. We note, however, that most genomes of subsection V cyanobacteria have draft genome status in NCBI’s RefSeq (contig or scaffold state), hence the classification of pseudogenes in these genomes remains to be verified. Additional genes have been reported to code for proteins involved in natural competence in specific cyanobacterial taxa (e.g., minor pilins [17, 23]). Since these genes are neither conserved nor essential for natural competence in other cyanobacteria, we considered these genes as accessory natural competence genes and excluded them from our analysis. Similarly, we did not consider here genes coding for transcriptional regulators involved in the regulation of NCG expression (e.g., sigma factor SigF/SigF2 [17, 24]) as core NCGs.

10.1128/msphere.00997-21.1 FIG S1 Protein sequence similarity among homologous NCGs within the subsection V cyanobacteria. Inside the group of subsection V cyanobacteria protein sequence similarity of NCGs homologs is 70% or higher, except for the major pilin PilA1 (60%). The standard error of the means revealed a maximum value of ±0.01% mean pairwise identities. Download FIG S1, EPS file, 0.1 MB. Copyright © 2022 Nies et al. 2022 Nies et al. https://creativecommons.org/licenses/by/4.0/ This content is distributed under the terms of the Creative Commons Attribution 4.0 International license.

10.1128/msphere.00997-21.4 TABLE S1 Coding sequence of core natural competence genes in *Synechocystis* sp. PCC 6803 and Synechococcus elongatus PCC 7942. NCBI accession numbers for the protein sequences used in this study are given. For all listed proteins, except RecA, in *Synechocystis* sp. PCC 6803 and/or Synechococcus elongatus PCC 7942, it was demonstrated that the respective mutants are not naturally transformable (14–20). RecA is essential for the post-DNA uptake recombination process and is considered ubiquitous in nearly all bacteria and was therefore used as a control for our analysis (38). Download Table S1, DOCX file, 0.01 MB. Copyright © 2022 Nies et al. 2022 Nies et al. https://creativecommons.org/licenses/by/4.0/ This content is distributed under the terms of the Creative Commons Attribution 4.0 International license.

**FIG 2:**
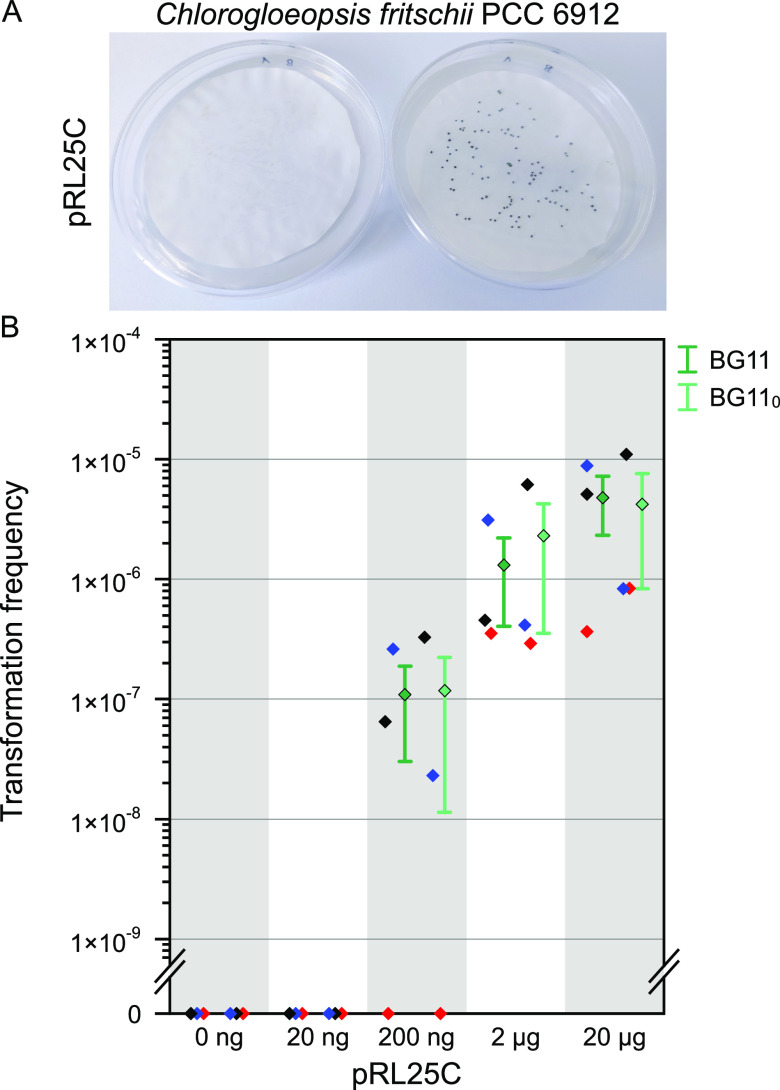
Natural transformation of Chlorogloeopsis fritschii PCC 6912. (A) Selection of *Chlorogloeopsis* colonies on nitrocellulose filters. A representative image showing either no transformants on selective BG11 plates after transformation without DNA or 20 ng of pRL25C (left) or Nm-resistant transformants after transformation with larger amounts of pRL25C (right). (B) Transformation frequencies of *Chlorogloeopsis* with plasmid pRL25C. Transformation frequency is given as the ratio of CFU with antibiotic selection (indicating transformation) to the number of CFU without antibiotic selection. The plot shows the dependency of the transformation frequency on the amount of introduced plasmid pRL25C (0 ng, 20 ng, 200 ng, 2 μg, or 20 μg) and the availability of combined nitrogen before selection (BG11 with 1.5 g/L NaNO_3_ and BG11_0_ without nitrate). Whiskers show one standard error of the mean. Single transformation frequencies from three independent experiments are given as single data points (red, blue, black).

To validate the computational prediction, we tested for the natural competence state by developing a natural transformation protocol for Chlorogloeopsis fritschii PCC 6912 (hereafter *Chlorogloeopsis*). As a member of subsection V cyanobacteria, *Chlorogloeopsis* is typically surrounded by a thick exopolysaccharide sheet (22). Unlike other true-branching subsection V cyanobacteria, *Chlorogloeopsis* shows filamentous growth only in early growth phases, divides in later growth phases in more than one plane, and eventually loses its filamentous structure (22). DNA transfer of the replicating plasmid pRL25C (providing kanamycin and neomycin [Nm] resistance, [25]) into *Chlorogloeopsis* was previously reported to be possible via conjugation and DNA bombardment (26, 27). Therefore, we decided to use that plasmid to test for natural competence in *Chlorogloeopsis*. Using a newly designed natural transformation protocol (see Materials and Methods), we were able to select Nm-resistant *Chlorogloeopsis* colonies that were naturally transformed with pRL25C (Fig. 2A). The presence of pRL25C in the resistant transformants was further validated by subsequent cultivation in antibiotic-supplemented suspension culture. Additionally, we confirmed the presence of the entire pRL25C plasmid in several individual suspension culture clones by polymerase chain reaction (PCR) using primers that amplify different regions of pRL25C (Fig. S2). Hence, we conclude that *Chlorogloeopsis* is naturally competent and that the whole pRL25C plasmid was reconstituted in *Chlorogloeopsis*.

10.1128/msphere.00997-21.2 FIG S2 Validation of Chlorogloeopsis fritschii PCC 6912 transformation with pRL25C by PCR. (A) PCR for plasmid presence. PCR products with primer pair Cf2/Cf3 from *Chlorogloeopsis* grown in suspension cultures, which were inoculated from randomly picked colonies from plates for every given transformation frequency given in Fig. 2, as well as a *Chlorogloeopsis* wild-type control (WT). pRL25C can be identified in transformants of each experiment but not in the WT. (B) PCR for plasmid integrity. PCR products of randomly picked colonies for one sample of the three independent experiments shown in Fig. 2 (1-1, 2-1, and 3-1) with the Cf primers as listed below. The second row shows the control reaction with *Chlorogloeopsis* WT and plasmid pRL25C with the Cf primers. Additionally, a PCR control with primers to amplify the 16S rRNA gene is shown for cell samples 1-1, 2-1, and 3-1, as well as for the WT. The Cf primer pairs cover the complete plasmid pRL25C. Primer pairs: Cf0/Cf1, Cf2/Cf3, Cf4/Cf5, Cf6/Cf7; primers for 16S rRNA: 27F1/1494Rc (30). Primer sequences are given in the Materials and Methods section. Download FIG S2, EPS file, 0.9 MB. Copyright © 2022 Nies et al. 2022 Nies et al. https://creativecommons.org/licenses/by/4.0/ This content is distributed under the terms of the Creative Commons Attribution 4.0 International license.

To evaluate the amount of DNA required for *Chlorogloeopsis* natural transformation, we quantified the transformation frequency in the presence and absence of combined nitrogen (BG11 and BG11_0_ media, respectively). The cultures were supplied with 20 ng to 20 μg of pRL25C, and the transformation frequency was determined by the ratio of colony forming units (CFU) growing on selective agar plates to the total number of CFU in the culture. Transformants were observed with DNA amounts of 200 ng of pRL25C and higher (Fig. 2B). Note that supplying the culture with 200 ng of pRL25C yielded transformants in only two of the three experiments, indicating that this concentration might be the lower threshold for the successful transformation of *Chlorogloeopsis* with pRL25C. Supplying the cultures with 2 μg or 20 μg of pRL25C yielded an increased transformation frequency between 2 × 10^−7^ and 2 × 10^−5^ with no significant effect of the supplied plasmid quantity (*P* = 0.55, using a Kruskal-Wallis test).

To test whether transformation efficiency is affected by nitrogen starvation, with the idea that DNA can serve as a nitrogen-containing nutrient, we compared natural competence frequencies of cells grown with or without combined nitrogen (that is, cells grown in BG11 or BG11_0_, respectively). We found that the presence of combined nitrogen in the growth media before transformation did not have an apparent effect on transformation frequency for the tested conditions (Fig. 2B).

The distribution of core NCGs in subsection V cyanobacteria suggests that other members could also be naturally competent. Yet, we note that the presence of NCGs alone does not always correspond to the presence of natural competence or that natural transformation can be demonstrated in the laboratory. For example, the genome of *Cyanothec*e sp. ATCC 51142 includes all NCGs; however, it was reported to be not naturally transformable (28). Besides the complete set of functional NCGs, natural competence depends on the expression dynamics of the involved proteins and can also depend on the environmental conditions. For example, the regulation of natural competence in Synechococcus elongatus PCC 7942 is influenced by the circadian clock. There, transformation efficiency is the lowest around dawn and the highest around dusk (17). Additionally, it cannot be excluded that additional, unknown genes involved in natural competence exist, and these genes are missing in our present analysis. Consequently, the presence of the NCGs, as derived from our bioinformatic predictions, is a necessary prerequisite for natural transformation; however, for sufficient validation of natural competence, it has to be verified experimentally.

Here, we report the robust DNA transfer into a member of subsection V cyanobacteria (*Chlorogloeopsis*) via natural transformation. While gene transfer into *Chlorogloeopsis* was already possible via conjugation (26, 27) and while the subsequent generation of knockout mutants by homologous recombination into the chromosome has been demonstrated (29), gene transfer by transformation could have some experimental advantages. For example, using gene delivery via conjugal transfer with Escherichia coli donors requires a further step to separate the donor cells from the recipient *Chlorogloeopsis* cells, and this step is not required during transformation.

Previous studies of other cyanobacteria demonstrated the role of genetic recombination in species diversification (30, 31) and in the evolution of novel phenotypic traits (32). The widespread presence of natural competence genes in cyanobacteria, including those in subsection V, may suggest that natural transformation is a substantial DNA acquisition mechanism in that phylum.

## MATERIALS AND METHODS

### Computational analysis.

Amino acid sequences of the core natural competence genes in *Synechocystis* sp. PCC 6803 were retrieved from the RefSeq database (33). Homologs to the core NCGs were searched by sequence similarity using BLAST (34) against 58 subject genomes. The amino acid sequence similarity between *Synechocystis* sp. PCC 6803 genes and the homologs in subsection V cyanobacteria was calculated using Needle (35), and tBLASTn was performed between subject genomes and *Synechocystis* sp. PCC 6803 natural competence genes (Fig. 1) if they revealed a sequence similarity below 25%. The pairwise protein sequence identities among the NCG homologs in subsection V cyanobacteria were calculated from a multiple sequence alignment reconstructed with a Multiple Alignment using Fast Fourier Transform (MAFFT) (36), using an in-house Python script.

### Growth and culture conditions.

Chlorogloeopsis fritschii PCC 6912 was obtained from the Pasteur Culture Collection of Cyanobacteria (PCC; Paris, France). *Chlorogloeopsis* cultures were grown photoautotrophically in BG11 medium or in a medium without combined nitrogen (BG11_0_) at 30°C and 30 μmol photon m^−2^ s^−1^ constant light. Suspension cultures of *Chlorogloeopsis* were grown in 50 mL media in a 300 mL Erlenmeyer flask at 100 rpm or in 15 mL media in a standing 50 mL cell culture flask at 150 rpm. For the selection of cyanobacterial transformants, BG11 plates were supplemented with 30 μg/mL Nm (Nm30). Note that *Chlorogloeopsis* wild-type cells cannot be cultivated on BG11 plates or in BG11 suspension media with 10 μg/mL Nm or higher.

### Plasmids used in this study.

The pDU1-based pRL25C plasmid (Nm resistance; [25]) was kindly provided by Enrique Flores (University of Sevilla, Spain). The pRL25C plasmid was isolated from the Escherichia coli XL1-Blue strain via standard plasmid DNA midi prep protocol.

### Natural transformation of *Chlorogloeopsis*.

50 mL of *Chlorogloeopsis* cultures were cultivated until an OD_750 nm_ of 0.3 to 0.37 in BG11 or BG11_0_. One day before transformation, the medium was refreshed via centrifugation of the suspension culture at 4,800 × *g* for 10 min at room temperature in 2 × 50 mL Falcon tubes per culture volume. The supernatant was removed, and the cells were resuspended in 50 mL of fresh medium of the same kind. After 1 d of cultivation, the cells were spun down again, most of the supernatant was removed, and the cells were resuspended in 1 to 2 mL of the remaining supernatant. The concentrated cell suspension was then split equally into 1.5 mL reaction tubes. Note that *Chlorogloeopsis* cultures sediment quickly and form aggregates, which can then be resuspended by repeated pipetting. Thoroughly mixing by pipetting is thus necessary directly before any step which requires equally mixed cultures, such as OD_750 nm_ measurement. The plasmid DNA of pRL25C (0 ng, 20 ng, 200 ng, 2 μg, 20 μg) was added to the concentrated cells and mixed. Cells and DNA were incubated in 1.5 mL reaction tubes for 1 to 2 h and mixed every 15 to 30 min by inverting. Afterwards, the cyanobacteria-DNA mixture was transferred to 15 mL of fresh medium (BG11 or BG11_0_) and cultivated for 2 d at standard growth conditions. Subsequently, the cell suspension was concentrated as before in 50 mL Falcon tubes, transferred to 1.5 mL reaction tubes, and concentrated further by centrifugation for 3 min at 4,800 × *g* at room temperature to a final volume of 300 μL. Then, 30 μL from each reaction tube was used to determine the average CFU via the plating of 200 μL of a 10^−5^ dilution on BG11 plates without antibiotics. The remaining cell suspension was plated on sterile nitrocellulose membranes placed on top of BG11 plates supplemented with Nm30. Plates were cultivated until colonies could be identified (up to one month). We note that although *Chlorogloeopsis* is motile (20), it does not move considerably on nitrocellulose filters, thereby allowing for the convenient detection of single colonies. To reliably maintain selective pressure, nitrocellulose membranes were transferred to fresh Nm30-containing plates every 14 days. The selection of colonies was verified by microscopy to exclude false-positive colonies (Fig. S3). After selection, randomly picked colonies were transferred into BG11 suspension culture supplemented with Nm30 to validate transformation-mediated resistance against Nm. Additionally, the uptake of DNA was tested via PCR on suspension cultures with specific primers.

10.1128/msphere.00997-21.3 FIG S3 Light microscopy pictures of *Chlorogloeopsis* during selection on BG11 plates supplemented with Nm. (A) Colony of *Chlorogloeopsis*. Intense dark green color and sharp distinction between colony and background is characteristic. (B and C) Examples of dying *Chlorogloeopsis* cells and cell debris, which somewhat resemble live *Chlorogloeopsis* colonies on nitrocellulose membranes but can be clearly distinguished from actual colonies by microscopy. Magnification: 100x. Context: *Chlorogloeopsis* forms aggregates in suspension culture, which can be partially but not completely resuspended by pipetting. Also, the intensity of aggregates depends on the growth phase of *Chlorogloeopsis*. As a consequence of this phenomenon, *Chlorogloeopsis* cultures on nitrocellulose filters on BG11 plates form aggregates and cannot be spread on plates as homogenously as other model species, such as Escherichia coli or *Synechocystis* sp. PCC 6803. In our experiments, selected colonies, which survived prolonged selection with Nm over one month, were verified as transformed colonies. However, for unexperienced observers it can be difficult, especially in the earlier stages of the selection process, to differentiate between actual colonies and aggregates of dying cells and cell debris. Also, *Chlorogloeopsis* colonies can be identified earlier via microscopy and the duration of the selection process can thus be shortened. Download FIG S3, EPS file, 2.7 MB. Copyright © 2022 Nies et al. 2022 Nies et al. https://creativecommons.org/licenses/by/4.0/ This content is distributed under the terms of the Creative Commons Attribution 4.0 International license.

### PCR verification of *Chlorogloeopsis* transformation.

100 μL of *Chlorogloeopsis* culture were frozen at −80°C, incubated for 15 min at 95°C, and crushed with a micro pestle. Cell lysate sample was further mixed and diluted until the color almost disappeared. Of this suspension, 1 μL was used as a DNA template for PCR with DreamTaq polymerase (ThermoFisher Scientific, Waltham, MA, USA) according to the manufacturer’s instructions. The primer pairs used to detect pRL25C were Cf0 (GTCAAACGTTGATCATGCAACAC) and Cf1 (GGCTTTCAACAGAAAGCATACG), Cf2 (CTGAGTTCGTCGGAGGTGAG) and Cf3 (GGGAGCTGCATGTGTCAGAG), Cf4 (GAATCGCTTCACGACCACG) and Cf5 (GCCAACCAATAAAGGGAGAATAGC), and Cf6 (GCTCGGTTAGCAATCCATTGG) and Cf7 (GCTTCGGTTAAGTAGCATAACAACC). As a positive control, primers 27F1 (AGAGTTTGATCCTGGCTCAG) and 1494Rc (TACGGCTACCTTGTTACGAC) for the 16S rRNA gene were used (37).

10.1128/msphere.00997-21.5 DATA SET S1 Table A: Amino acid sequence similarity between the core NCGs of *Synechocystis* sp. PCC 6803 genes and the reciprocal best hits of 58 cyanobacterial genomes of subsection V cyanobacteria. Table B: Assembly accession numbers of the cyanobacterial subject genomes that have been used to find homologs of the NCGs of *Synechocystis* sp. PCC 6803 in subsection V cyanobacteria. The protein accessions of the reciprocal best hits in the subject genomes are listed. Table C: tBLASTn results between the NCGs of *Synechocystis* sp. PCC 6803 and the subject genomes that revealed sequence similarities below 25% in Fig. 1. Download Data Set S1, XLSX file, 0.03 MB. Copyright © 2022 Nies et al. 2022 Nies et al. https://creativecommons.org/licenses/by/4.0/ This content is distributed under the terms of the Creative Commons Attribution 4.0 International license.
