# Supplementary material for: Natural Competence in the Filamentous, Heterocystous Cyanobacterium Chlorogloeopsis fritschii PCC 6912
Source: mSphere. 2022 Jul 14;7(4):e00997-21. doi: 10.1128/msphere.00997-21 (PMC9429965; doi:10.1128/msphere.00997-21)
Supplement: TABLE S1 [file msphere.00997-21-st001.docx]

| NCGs | *Synechocystis* sp. PCC 6803 | *Synechococcus elongatus* PCC 7942 |
| --- | --- | --- |
| PilA1 | WP_010872381.1 | WP_011377419.1 |
| PilB1 | WP_010873519.1 | WP_011378301.1 |
| PilC | WP_014407124.1 | WP_011378299.1 |
| PilD | WP_010871270.1 | WP_011244469.1 |
| PilM | WP_010872897.1 | WP_011243962.1 |
| PilN | WP_010872898.1 | WP_011378470.1 |
| PilO | WP_010872899.1 | WP_011378469.1 |
| PilP | WP_010872502.1 | WP_011377474.1 |
| PilQ | WP_010872900.1 | WP_011378468.1 |
| PilT1 | WP_010873187.1 | WP_011378300.1 |
| ComEA | WP_010873643.1 | WP_011377526.1 |
| ComEC | WP_010871755.1 | WP_011378473.1 |
| ComF | WP_010873338.1 | WP_011378447.1 |
| DprA | WP_010872006.1 | WP_011377689.1 |
| Hfq | WP_010872821.1 | WP_011244478.1 |
| EbsA | WP_010874024.1 | WP_011242990.1 |
| RecA | WP_010874337.1 | WP_011243477.1 |
